# Molecular Evolution and Functional Divergence of the Ca^2+^ Sensor Protein in Store-operated Ca^2+^ Entry: Stromal Interaction Molecule

**DOI:** 10.1371/journal.pone.0000609

**Published:** 2007-07-11

**Authors:** Xinjiang Cai

**Affiliations:** Department of Cell Biology, Duke University Medical Center, Durham, North Carolina, United States of America; Laboratory of Neurogenetics, National Institutes of Health, United States of America

## Abstract

Receptor-mediated Ca^2+^ signaling in many non-excitable cells initially induces Ca^2+^ release from intracellular Ca^2+^ stores, followed by Ca^2+^ influx across the plasma membrane. Recent findings have suggested that stromal interaction molecules (STIMs) function as the Ca^2+^ sensor to detect changes of Ca^2+^ content in the intracellular Ca^2+^ stores. Human STIMs and invertebrate STIM share several functionally important protein domains, but diverge significantly in the C-terminus. To better understand the evolutionary significance of STIM activity, phylogenetic analysis of the STIM protein family was conducted after extensive database searching. Results from phylogeny and sequence analysis revealed early adaptation of the C-terminal divergent domains in Urochordata, before the expansion of STIMs in Vertebrata. STIMs were subsequently subjected to one round of gene duplication as early as in the Euteleostomi lineage in vertebrates, with a second round of fish-specific gene duplication. After duplication, STIM-1 and STIM-2 molecules appeared to have undergone purifying selection indicating strong evolutionary constraints within each group. Furthermore, sequence analysis of the EF-hand Ca^2+^ binding domain and the SAM domain, together with functional divergence studies, identified critical regions/residues likely underlying functional changes, and provided evidence for the hypothesis that STIM-1 and STIM-2 might have developed distinct functional properties after duplication.

## Introduction

In response to appropriate stimuli, virtually all types of animal cells can initiate spatial and temporal changes of cytosolic free Ca^2+^ concentrations to regulate a wide range of physiological processes [1]. Accordingly, animal cells employ a repertoire of membrane transporters such as Ca^2+^ channels, Ca^2+^ ATPases, and cation/Ca^2+^ exchangers to control cytosolic Ca^2+^ [2], [3]. One important mode of Ca^2+^ influx across the plasma membrane involves Ca^2+^ release from intracellular Ca^2+^ store through the inositol 1,4,5-trisphosphate receptors, followed by activation of store-operated Ca^2+^ (SOC) channels [4]. SOC currents, for instance, the Ca^2+^ release activated Ca^2+^ (CRAC) current (I*_CRAC_*), have been well characterized biophysically and pharmacologically. However, the molecular identities of SOC entry have remained elusive for almost two decades [4].

Within the last two years, accumulating evidence suggests that stromal interaction molecules (STIMs) and Orai proteins might act as the Ca^2+^ sensor in the intracellular Ca^2+^ store and the putative CRAC channel at the plasma membrane, respectively [5]–[7] (but also see reference [8]). Overexpression of both STIMs and Orai molecules generate currents that recapitulate the properties of I*_CRAC_* [9]–[11]. STIMs are single-span membrane proteins [12] with an unpaired N-terminal EF-hand Ca^2+^ binding domain critical for the Ca^2+^ sensor function [13]–[15]. In addition, STIMs contain an N-terminal sterile α motif (SAM) domain and a C-terminal (cytoplasmic) coiled-coil/ERM domain [12]. Following intracellular Ca^2+^ store depletion, STIM-1 accumulates and redistributes at distinct membrane regions, and then leads to activation of I*_CRAC_* [15], [16]. Mutations of conserved acidic residues in the EF-hand domain of STIM-1, which presumably reduce its Ca^2+^ affinity, mimic the Ca^2+^ store depletion phenomenon with constitutively active SOC entry [14], [15]. Furthermore, *in vitro* studies also suggest that the N-terminal EF-hand and SAM region of human STIM-1 exists as monomers when binding to Ca^2+^, but readily undergoes oligomerization in the Ca^2+^-depleted state [17]. Taken together, compelling evidence has suggested that STIM-1 functions as the Ca^2+^ store sensor in SOC entry. In contrast, the role of the closely related STIM-2 protein in regulating I*_CRAC_* is less defined [13], [15], [18]. In response to Ca^2+^ store depletion, STIM-2 may behave differently and negatively regulate STIM-1-induced SOC entry [18].

To further understand the significance of I*_CRAC_* in regulating many cellular functions, it is important to define the molecular and cellular mechanisms for STIM-mediated activation of CRAC channels. Evolutionary analysis can provide useful guides for molecular, biophysical, and biochemical analyses of functional and regulatory mechanisms of ion channels and transporters [19], as shown in our previous work on the phylogeny and structural analysis of the cation/Ca^2+^ exchangers [2] and the membrane protein adaptor molecule ankyrin [20]. Our recent report on the Orai protein family, the putative CRAC channel subunit, has also provided novel insights into our understanding of the evolution and structural domains of Orai proteins [3].

In the present work, I have applied rigorous evolutionary and bioinformatics analysis to (1) elucidate the evolutionary history and gene duplication events in the STIM protein family by extensive database searching and constructions of phylogenetic trees; (2) identify potential sequence determinants underlying functional divergence of STIM proteins after gene duplication by mapping specific residues onto the STIM protein domains and detecting putative residues subjected to distinct selective evolutionary constraints with maximum likelihood estimates. Functional significance of these findings will also be discussed in relation to applying evolutionary information to structure and function studies of STIM proteins.

## Results and Discussion

### Duplication of the STIM Protein Family During Chordate Evolution

Identification and characterization of STIM molecules as an essential component mediating I*_CRAC_* in *Caenorhabditis elegans* (STIM-1) [21], [22], *Drosophila melanogaster* (STIM-1) and *Homo sapiens* (STIM-1 and -2) [13], [15] suggest that STIM proteins are evolutionarily conserved across metazoans. However, a comprehensive analysis of the phylogenetic relationship of the STIM protein family is important to understand results from biological experiments in terms of evolutionary significance, such as the evolution of critical protein domains and functional divergence of duplicated gene products, among others.

**Figure 1:**
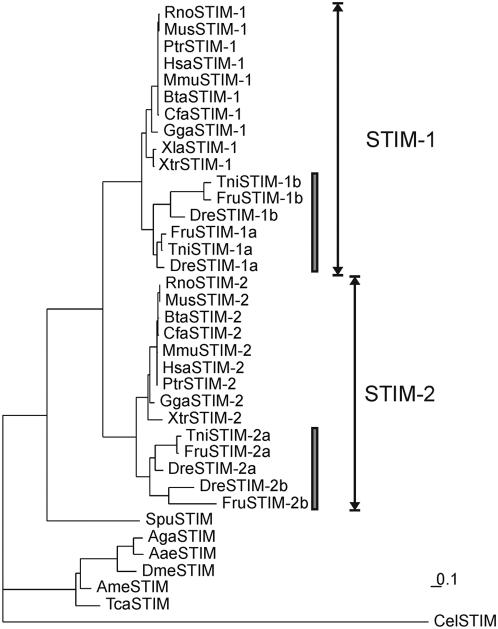
Maximum likelihood tree of the STIM protein family. The phylogenetic tree constructed with the program PROML [45] shows the evolutionary relationship of the STIM protein family. The two vertebrate branches, STIM-1 and STIM-2, are indicated with *black arrows*. Fish-specific duplications in vertebrate STIM branches are indicated with *black bars*. Sequences that failed in the 5% chi-square test of Tree Puzzle [44] were removed for further phylogenetic analysis (Table S1). The unit of branch length is the expected fraction of amino acids substitution. Aae, *A. aegypti*; Aga, *A. gambiae*; Ame, *A. mellifera*; Bta, *B. Taurus*; Cel, *C. elegans*; Cfa, *C. familiaris*; Dme, *D. melanogaster*; Dre, *D. rerio*; Fru, *F. rubripes*; Gga, *G. gallus*; Hsa, *H. sapiens*; Mmu, *M. mulatta*; Mus, *M. musculus*; Ptr, *P. troglodytes*; Rno, *R. norvegicus*; Spu, *S. purpuratus*; Tca, *T. castaneum*; Tni, *T. nigroviridis*; Xla, *X. laevis*; Xtr, *X. tropicalis*.

**Figure 2:**
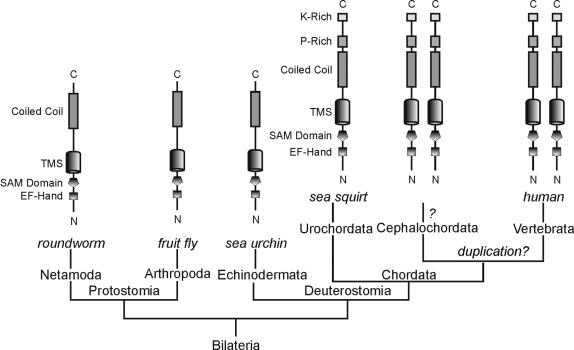
Evolution of the protein domains in the STIM protein family. Schematic representations of protein domains of representative STIM molecules based on phylogeny of bilaterian animals [51]. The proline-rich domain and the lysine-rich domain of STIM molecules, which are absent in worms, insects and sea urchin, first appeared in Urochordata, as shown in *C. intestinalis*. Duplication of STIM molecules speculated to have occurred after Cephalochordata/Urochordata divergence is indicated with “*?*”. Fish-specific duplication of STIMs (Fig. 1) is not illustrated here. *C*, C-terminus; *K-rich*, lysine-rich domain; *N*, N-terminus; *P-rich*, proline-rich domain; *TMS*, transmembrane segment.

Here, by extensive database searching, 40 nonredundant STIM sequences were identified from 22 species analyzed in this study (Table S1 in Supplementary Information), including sequences from Echinodermata *Strongylocentrotus purpuratus*, Urochordata *Ciona intestinalis*, and several nonmammalian Vertebrata species. Phylogenetic trees constructed with maximum likelihood (Fig. 1), maximum parsimony, and neighbor-joining methods as well as weighted neighbor-joining analysis (data not shown) were compared to infer the congruent phylogeny. The identical overall tree topologies generated from these different molecular phylogenetic approaches indicated consistent and reliable results for evolutionary relationships of the STIM protein family. Homologues of STIM-1 and -2 are present as early as in bony fishes (*Takifugu rubripes, Tetraodon nigroviridis, Danio rerio*), while *C. intestinalis* and *S. purpuratus* appear to contain only one copy of STIM molecule in each genome (Figs. 1 and 2, and Table S1). *C. intestinalis* has been shown to comprise single copies of many vertebrate gene families [20], [23] likely arising from hypothesized large-scale genome duplications in vertebrates diverging from Urochordata and Cephalochordata [24]. Thus, gene duplication of the STIM family appeared to have occurred as early as in Euteleostomi lineage to give rise to the two major STIM branches in vertebrates. Orai molecules also underwent a round of gene duplication at the early stage of vertebrates [3]. Duplication of STIM and Orai proteins, and possibly, other subunits, might have evolved to fit the adaptation of SOC entry into more advanced vertebrate physiology.

**Figure 3:**
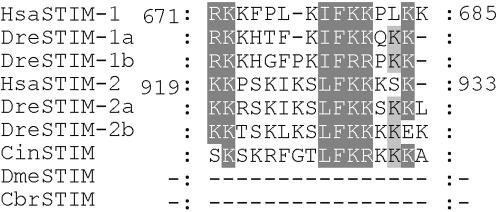
Sequence alignment of the lysine-rich domain from representative species. Shown is the sequence alignment of the lysine-rich domain of STIM molecules, which are numbered according to human STIM-1 and STIM-2.

**Figure 4:**
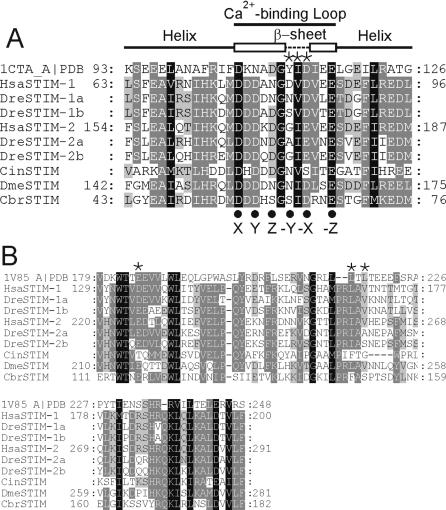
Structural analysis of the EF-hand domain and the SAM domain of STIMs. A. Sequence alignment of the EF-hand domain of representative STIM proteins and the chicken troponin C site III sequence (PDB entry 1CTA). The structural characteristics of 1CTA including two α-helices and the central Ca^2+^-binding loop are indicated above the sequence alignment. The positions of Ca^2+^ binding ligands are indicated with *dark circle* symbols below the alignment. The three residues of the β-sheet structure are marked with *asterisk* symbols. B. Sequence alignment of the SAM domain of representative STIM proteins and PDB entry 1V85. The two hydrophobic residues (Leu216 and Leu218 in IV85) at the oligomeric interface of SAM domains are indicated with *asterisk* symbols. One acidic residue (Glu185 in IV85) possibly affecting the aggregation state of SAM domains [39] is also indicated.

In fish genomes, STIM underwent a second round of duplications that resulted in, as far as can be ascertained in the current database, four copies of STIM molecules in each fish genome (Fig. 1 and Table S1). The fish-specific genome duplication is speculated to have occurred approximately 350 million years ago, after splitting from other vertebrates [25]. While possibly due to functional redundancy, most fish-specific duplicated genes were subsequently lost, the remaining duplicated genes might have evolved new functions. Indeed, sequence analysis of these duplicated fish STIMs reveals substantial sequence divergence even in the conserved protein domains (Figs. 3 and 4). The Orai protein family also exhibits fish-specific duplications in the Orai-1 group [3]. It remains intriguing if duplicated Orai and STIM molecules specific in the fish genomes cooperate to perform as yet uncharacterized, novel functions.

Invertebrate STIMs, human STIM-1 and STIM-2 share several highly conserved protein domains including the EF-hand Ca^2+^-binding domain, the SAM domain, and the coiled-coil/ERM domain, but diverge remarkably C-terminal to the coiled-coil/ERM domain [12]. Human STIM molecules contain a proline-rich domain and a lysine-rich tail domain at the C-terminus (Fig. 2). The C-terminal lysine-rich region of human STIM-1, but not the proline-rich domain, has been demonstrated to be involved in activating CRAC channels in mammalian cells [26]. It has been hypothesized that the lysine-rich-domain-mediated regulation of CRAC channels is a vertebrate-specific adaptation [26]. Analysis conducted in this study, however, reveals the presence of the proline-rich domain and the lysine-rich domain in *C. intestinalis*, suggesting early adaptation of these two domains in Urochordata before the expansion of STIMs in vertebrates (Fig. 2). Fig. 3 shows the sequence alignment of the C-terminal lysine-rich regions from representative species. The lysine-rich domain is absent in STIM of Nematoda, Arthropoda and Echinodermata. Therefore, the primordial form of vertebrate STIMs with all the conserved protein domain structures seemed to have evolved in *C. intestinalis* during early Chordata evolution. We also made similar observations on the evolution of the Orai protein family [3]. The single copy of Orai protein in *C. intestinalis* also contains similar domain structures of vertebrate Orai molecules. Thus, STIM and Orai in Urochordata might have evolved to establish the primordial functional properties of vertebrate-type I*_CRAC_*. I*_CRAC_* detected in mammals, presumably representing the vertebrate-type I*_CRAC_*, displays similar biophysical properties with I*_CRAC_* observed in *D. melanogaster* S2 cells, but differs in their inactivation properties [27]. Their different inactivation properties have been proposed to correlate with the adaptation of the lysine-rich domain in Chordata, and possibly involve interaction between the lysine-rich domain of STIMs and a stretch of basic residues immediately preceding the first transmembrane segment of Orai proteins [28]. In addition, SOC currents measured in the *C. elegans* intestinal cells and HEK cells overexpressing *C. elegans* STIM and Orai homologues also exhibit some distinct properties despite many similarities with mammalian I*_CRAC_* [29]. It remains to be determined whether the functional properties of SOC currents in *C. intestinalis* would be more similar to mammalian I*_CRAC_* than those of SOC currents observed in Protostomes.

The Ka/Ks ratios are often used to detect positive selective pressures over the coding sequence of a gene [30]. The ratios calculated between members of STIM-1 or between members of STIM-2 are much less than 1, such as 0.1775 for HsaSTIM-1 (human) vs. XtrSTIM-1 (frog) and 0.0510 for HsaSTIM-1 (human) vs. MusSTIM-1 (mouse), suggesting strong purifying selections (evolutionary constraints) within each vertebrate STIM group. Therefore, although STIM-1 and -2 diverged after duplication, their individual functional properties might have been highly conserved across vertebrate species.

### Evolution of the EF-hand and SAM domains with Structural Correlations

To gain novel insights into the evolutionary perspective on the structure and function relationships of STIMs, I unitized two complementary approaches: (1) mapping potentially critical residues by sequence alignments of STIM structural domains across representative species; (2) detecting phylogeny-based site-specific evolutionary rate changes with the program DIVERGE [31]. Duplication of STIM molecules only occurred in vertebrates. Thus, I will focus subsequent evolution and divergence analysis on vertebrate STIM molecules.

The EF-hand and SAM domains at the N-terminus of STIMs are highly conserved across metazoans (Figs. 4A and 4B) [12]. EF-hand domain is believed to function as the Ca^2+^ sensor in the intracellular Ca^2+^ stores [14], [15] while SAM domain is thought to mediate protein-protein interactions of STIM proteins [17]. STIMs contain an unpaired EF-hand domain. Although EF-hand domains usually occur as pairs in proteins, this problem could be resolved by oligomerization of STIM molecules [12], [17]. The consensus EF-hand sequence contains about 6 to 7 Ca^2+^ ligands coordinated in a 12-residue Ca^2+^ binding loop, which usually starts with an Asp and ends with a Glu [32]. The main-chain carbonyl oxygen atom of the central residue (*-Y* position) in the Ca^2+^ binding loop contributes as one Ca^2+^ ligand, followed immediately by a hydrophobic amino acid (usually, Ile, Val or Leu) and another residue at the *–X* position. These three residues (i.e. Tyr-Ile-Asp in PDB entry 1CTA, Fig. 4A) [33] form a short β-sheet structure linking the Ca^2+^-binding loops with the side chain of the hydrophobic residue positioned in the center of the hydrophobic core of the EF-hand domain. The β-sheet structure is proposed to orchestrate the conformational changes of the N-terminal and the C-terminal parts of the Ca^2+^-binding loop upon Ca^2+^ binding, and induce coordinated movements of the two α helices surrounding the Ca^2+^-binding loop and the resultant overall conformational changes of the EF-hand domain [32].

The chicken troponin C site III structure (PDB entry 1CTA, chain A) [33] emerged as a significant hit to the EF-hand domain sequences of human STIM-1 and -2 during similarity searches of the SWISS-Model server [34] or the PDB database [35]. Alignment of the EF-hand domain of representative STIM molecules reveals the highly conserved residues required for Ca^2+^ ligand binding, compared with the troponin C site III (Fig. 4A) and other EF-hand domain-containing sequences (data not shown). STIM molecules possess the conserved EF-hand domain structure composed of the 12-residue Ca^2+^-binding loop and the two surrounding α-helices. Consistent with the role as the Ca^2+^ sensor, mutation of Ca^2+^ ligand residues in *Drosophila* STIM-1 and human STIM-1 causes constitutive activation of CRAC channels by mimicking Ca^2+^ store depletion [14], [15]. However, similar mutations in the human STIM-2 EF-hand domain do not appear to interfere with the observed inhibitory function of human STIM-2 [18], suggesting that the EF-hand domain in STIM-2 might not be important for STIM-2 inhibitory activity. It should be noted that such mutations might alter some unknown, yet uncharacterized, functional properties of STIM-2.

One noteworthy difference between human STIM-1 and -2, and other EF-hand domain sequences is the proposed β-sheet sequence (Fig. 4A) [32], [33]. The first residue (*-Y* position) of the β-sheet, whose main-chain carbonyl oxygen serves as a Ca^2+^ ligand, is most often Tyr/Phe/Thr in consensus EF-hand sequences. In all identified invertebrate STIMs and vertebrate STIM-1 molecules, the corresponding residue at the *–Y* position are Asn/Asp/Ser, compared with predominantly Gly in identified vertebrate STIM-2 proteins (except Ala in DreSTIM-2a). Gly is known to be an intrinsically destabilizing residue in β sheets [36], [37]. Whether this change to Gly in STIM-2 molecules plays a role in Ca^2+^-binding by affecting the coordinated conformational changes resulting from Ca^2+^ binding [32], as described above, remains to be established. In addition, the third residue (*-X* position) is Asp in most invertebrate STIMs and all vertebrate STIM-1, consistent with the consensus EF-hand domain sequence. In contrast, the corresponding residue in vertebrate STIM-2 is Glu, occurring less often at *–X* position.

**Figure 5:**
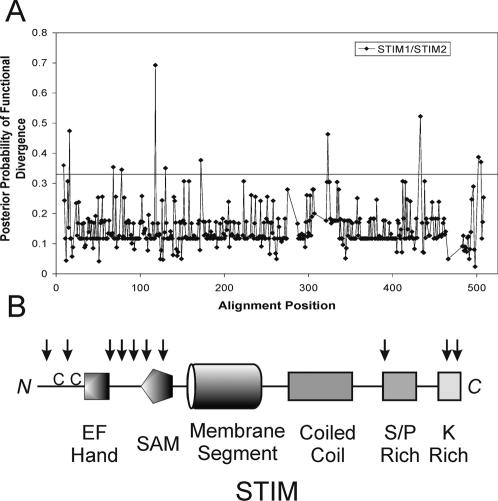
Sites with altered evolutionary rates identified by posterior probability of functional divergence. A. The posterior probability of functional divergence between vertebrate STIM-1 and STIM-2 branches were calculated with the program DIVERGE [31]. 11 of the aligned sites showed posterior probabilities >0.33. The 9th site fell in a less defined region and was excluded for further analysis. The neighbor-joining tree used for DIVERGE (Fig. S1) is available in Supplementary Information. B. The localization of the residues with significant posterior probabilities was mapped onto the structural model of vertebrate STIM molecules, indicated with *black arrows*. The highly conserved double cysteine-pair region is shown as a double “C” before the EF-hand domain in the N-terminus. *C*, C terminus; *N*, N terminus.

Nevertheless, the overall EF-domain sequences of STIM molecules, especially the Ca^2+^ ligands, appear to be similar to the consensus EF-domain Ca^2+^-binding sequence. Interestingly, functional divergence analysis did not identify any significant hit in the EF-hand domain region of vertebrate STIM branches. Instead, half (five out of ten) residues detected as high probability for functional divergence are located at the linker region between the EF and the SAM domain, or at the beginning of the SAM domain (see below, Fig. 5). Thus, an alternative explanation for the apparent failure of EF-hand mutations to affect the STIM-2 inhibitory action [18] may be less efficient transmission of Ca^2+^-binding induced conformational changes to the SAM domain and/or the cytoplasmic protein domains in STIM-2. Ca^2+^ binding causes structural changes of the Ca^2+^-binding loop and the coordinated movements of two α-helices [32], with the C-terminal helix connecting to the linker region. Indeed, in human STIM-1, Ca^2+^-binding or dissociation results in drastic global conformational changes in the recombinant EF-SAM region, not just limited to the EF-hand domain [17].

Next, the SAM domain sequences of representative STIM molecules were aligned and analyzed (Fig. 4B). The SAM domain is a ∼70-residue domain believed to mediate protein interactions in a wide range of signaling molecules [38]. *In vitro* biophysical characterization revealed Ca^2+^-dependent conformational changes and associated oligomerization in the human STIM-1 EF-SAM domain [17]. STIM molecules possess helix bundle arrangements similar to other SAM domains (data not shown). Two critical hydrophobic residues (aligned positions at L216 and L218 in PDB entry 1V85, marked in Fig. 4B) are believed to be thermodynamically important for SAM domain interactions to form the oligomeric state [39]. These two residues are located at the oligomeric interface of the SAM domain in many other proteins, and may reflect evolution from a common ancestor structure. Mutations of these two residues to Ala could significantly affect SAM domain interactions. These two residues are conserved as Leu and Val in STIM-1, and Ile and Val in most STIM-2 molecules, while not in fish STIM-2 proteins (Fig. 4B). The SAM domain of fish STIM-2a/b molecules might have evolved differently or land vertebrates regained the conserved module by convergent evolution after diverging from fish species.

### Phylogeny-based Functional Divergence Analysis of Vertebrate STIMs

Gene duplications provide a means to evolve novel biological functions, and changes in protein functions may then yield different evolutionary constraints on duplicated genes [40]. To gain evolutionary statistics on the functional divergence of the duplicated vertebrate STIMs, the two vertebrate STIM branches were analyzed with the program DIVERGE [31] to detect shifts in the evolutionary rates and identify amino acid residues likely responsible for functional divergence. This phylogeny-based method is expected to complement our approaches based on sequence alignments and structural analysis.

Estimation of the coefficient of functional divergence [31] (θ = 0.151) between vertebrate STIM-1 and -2 provides evidence for the hypothesis that these two vertebrate STIM branches (Fig. S1) underwent distinct evolutionary constraints and structural diversification. Interestingly, if the invertebrate STIMs were clustered as an independent group in the divergence analysis (with the exclusion of the C-terminal divergent domain sequences), there was no significant difference between STIM-1 and the invertebrate cluster (θ = 0.001). The functional divergence estimation between STIM-2 and the invertebrate cluster remained substantial (θ = 0.224). In line with evidence from our structural analysis above, the divergence analysis has again implicated that STIM-1 [5]–[7] might have maintained the basic functional properties of Protostomia STIMs [27], [29] in mediating SOC entry although acquirement of the C-terminal divergent domains might add some new regulatory properties [27] in response to new evolutionary pressure. In contrast, STIM-2 likely diverged to evolve new functional properties in vertebrates [18]. Knockdown of STIM-2 does not affect SOC entry and I*_CRAC_* in mammalian cells [13] and co-expression of STIM-2 and Orai-1 generates phenotypes similar to those of HEK cells expressing Orai-1 alone [11].

Posterior probability analysis of the program also identified residues likely contributing to the functional divergence (Fig. 5), 10 of which are calculated with significant posterior probabilities more than 0.33 in well-defined regions. The first two residues are located in the highly conserved cysteine-pair region [12] at the N-terminus, with each residue two spaces before corresponding cysteine residues. Three residues are present at the linker region between the EF-hand domain and the SAM domain with another two in the SAM domain, possibly involved in conformational changes at the EF-hand and SAM region in the presence or absence of Ca^2+^. One residue localizes to the S/P-rich region, with a potential Ser phosphorylation site only in human STIM1 (probability 0.50) (Met in human STIM-2). The remaining two residues are found in the lysine-rich domain. Overall, functional divergence analysis supports that STIM-1 and -2 diverged after gene duplication and displayed shifts in the evolutionary rates in specific residues.

In conclusion, this study has revealed detailed evolutionary history of the STIM protein family, an essential component of the Ca^2+^ release-activated Ca^2+^ entry. Urochordata appear to possess all the conserved protein domains of vertebrate STIM molecules, indicating that modern STIM structure/function is a Chordata adaptation. Subsequent gene duplication in vertebrates as early as in Euteleostomi lineage led to two distinct STIM branches. Evolutionary analysis supports the hypothesis from biophysical and physiological studies that STIM-1 and -2 might have evolved different functional properties, with changes in the evolutionary rates after duplication. This study, together with the recent report on molecular evolution of Orai proteins [3], present the first original and comprehensive bioinformatics studies of Ca^2+^-release activated Ca^2+^ entry by demonstrating biochemical and physiological significance of Ca^2+^-release activated Ca^2+^ entry in light of evolutionary origins, and therefore, provides novel evolutionary aspects of findings obtained from biochemical studies [5]–[7].

## Materials and Methods

### Preparation of Data Sets, Sequence Alignment and Phylogenetic Analysis

Sequence data sets of the STIM protein family were obtained by PSI-Blast and TBlastN searches [41] of the non-redundant protein databases at the National Center for Biotechnology Information (NCBI) and UniProt/SwissProt, and genomic databases at NCBI and Ensembl, respectively, using DmeSTIM (Accession Number AAK82338). Sequences were then processed essentially as previously described [2], [20]. Amino acid sequences were aligned with ClustalX (version 1.83) [42] and manually refined with GeneDoc (version 2.6) [43].

Sequences were subsequently exported as PHYLIP format and subjected to the 5% chi-square test implemented in the Tree-Puzzle (version 5.2) [44]. Phylogenetic trees were constructed with maximum likelihood (PROML), maximum parsimony (PROTPARS), and neighbor-joining (PROTDIST and NEIGHBOR) programs implemented in the PHYLIP program package (version 3.6) [45], essentially as previously described [2], [20]. Weighted neighbor-joining analysis were performed using WEIGHBOR [46] with distance matrix derived from Tree-Puzzle or PHYLIP programs.

The EF-hand domain sequence of STIMs was identified by consensus EF hand sequence searches [12]. The SAM domain region was predicted by the SMART (a Simple Modular Architecture Research Tool) server [47]. The resulting domain sequences of human STIM-1 and -2 were submitted to the SWISS-Model server [34] or the PDB database [35] to search for the best fitting structural models. Sequence alignment of STIM domain sequences from representative species and identified structural model sequences was performed with ClustalX and displayed with GeneDoc as described above.

### Calculation of the Ratio of Nonsynonymous (Ka) to Synonymous (Ks) Rate

The Ka/Ks ratios of selected STIM molecules were calculated with yn00 implemented in PAML (Version 3.15) [48]. mRNA sequences were retrieved from NCBI database and submitted to the PAL2NAL web server (http://coot.embl.de/pal2nal/) [49] along with related multiple protein sequence alignment. The PAL2NAL server first converts each protein sequence in the multiple sequence alignment into DNA sequences in a regular expression pattern, which are then compared with the input nucleotide sequences to search for the corresponding codon alignment. The resulting codon alignments in PAML format were then subjected to Ka/Ks ratio calculation with yn00.

### Functional Divergence and Site-specific Evolutionary Rate Estimates after Duplication

Site-specific changes in evolutionary rate after gene duplication (type I functional divergence) was estimated to detect functional divergence of a protein family [31], [50]. Maximum likelihood estimate for theta, the coefficient of functional divergence, was measured with the program DIVERGE (version 2.0) [31]. A *de novo* neighbor-joining tree was constructed with Poisson distance and re-rooted. Clusters of refined protein sequences were then selected for likelihood ratio tests and posterior site analysis.

## Supporting Information

Table S1 List of Proteins Used for Analyses
(0.06 MB DOC)

Figure S1 Neighbor-joining tree used for the program DIVERGE. The tree was constructed with Poisson distance matrix implemented in DIVERGE, and re-rooted with the invertebrate STIM sequence AmeSTIM. The two branches for STIM-1 and STIM-2 were then selected as corresponding clusters for subsequent functional divergence analysis between two branches.
(1.91 MB TIF)
