# Supplementary material for: Molecular Evolution and Functional Divergence of the Ca2+ Sensor Protein in Store-operated Ca2+ Entry: Stromal Interaction Molecule
Source: PLoS One. 2007 Jul 11;2(7):e609. doi: 10.1371/journal.pone.0000609 (PMC1904252; doi:10.1371/journal.pone.0000609)
Supplement: Table S1 — List of Proteins Used for Analyses (0.06 MB DOC) [file pone.0000609.s001.doc]

# Table S1. List of Proteins Used for Analyses

| **Name** | Organism | Common Name | **Taxonomy** | **Identifier** | Database |
| --- | --- | --- | --- | --- | --- |
| AaeSTIM | *Aedes aegypti* | yellow fever mosquito | Invt. | 108869895 | GenBank |
| AgaSTIM | *Anopheles gambiae* | malaria mosquito | Invt. | 58381040 | GenBank |
| AmeSTIM | *Apis mellifera* | honeybee | Invt. | 110757132 | GenBank |
| CbrSTIM | *Caenorhabditis briggsae* | roundworm | Invt. | 39588863 ***** | GenBank |
| CelSTIM | *Caenorhabditis elegans* | roundworm | Invt. | 110746956 | GenBank |
| DmeSTIM | *Drosophila melanogaster* | fruit fly | Invt. | 15042568 | GenBank |
| TcaSTIM | *Tribolium castaneum* | red flour beetle | Invt. | 91081005 | GenBank |
| SpuSTIM | *Strongylocentrotus purpuratus* | purple sea urchin | Invt. | 115973379 | GenBank |
| CinSTIM | *Ciona intestinalis* | sea squirt | Invt. | ENSCINP00000022039 ***** | Ensembl |
| DreSTIM-1a | Danio rerio | zebrafish | Vt. | 113677986 | GenBank |
| DreSTIM-1b | *Danio rerio* | zebrafish | Vt. | ENSDARP00000082360 | Ensembl |
| DreSTIM-2a | *Danio rerio* | zebrafish | Vt. | 68364168 | GenBank |
| DreSTIM-2b | Danio rerio | zebrafish | Vt. | 68354194 | GenBank |
| FruSTIM-1a | Fugu rubripes | Japanese pufferfish | Vt. | NEWSINFRUP00000152028 | Ensembl |
| FruSTIM-1b | *Fugu rubripes* | Japanese pufferfish | Vt. | NEWSINFRUP00000136714 | Ensembl |
| FruSTIM-2a | *Fugu rubripes* | Japanese pufferfish | Vt. | NEWSINFRUP00000133113 | Ensembl |
| FruSTIM-2b | *Fugu rubripes* | Japanese pufferfish | Vt. | NEWSINFRUP00000141041 | Ensembl |
| TniSTIM-1a | Tetraodon nigroviridis | green spotted pufferfish | Vt. | 47227834 | GenBank |
| TniSTIM-1b | *Tetraodon nigroviridis* | green spotted pufferfish | Vt. | 47211362 | GenBank |
| TniSTIM-2a | *Tetraodon nigroviridis* | green spotted pufferfish | Vt. | 47227425 | GenBank |
| TniSTIM-2b | *Tetraodon nigroviridis* | green spotted pufferfish | Vt. | GSTENT00002137001 ***** | Ensembl |
| XlaSTIM-1 | *Xenopus laevis* | African clawed frog | Vt. | 116487984 | GenBank |
| XtrSTIM-1 | *Xenopus tropicalis* | Western clawed frog | Vt. | 45433580 | GenBank |
| XtrSTIM-2 | *Xenopus tropicalis* | Western clawed frog | Vt. | 89266839 | GenBank |
| GgaSTIM-1 | *Gallus gallus* | chicken | Vt. | 71895083 | GenBank |
| GgaSTIM-2 | *Gallus gallus* | chicken | Vt. | ENSGALP00000023141 | Ensembl |
| MusSTIM-1 | *Mus musculus* | house mouse | Mam. | 31981983 | GenBank |
| MusSTIM-2 | *Mus musculus* | house mouse | Mam. | 50510951 | GenBank |
| RnoSTIM-1 | *Rattus norvegicus* | norway rat | Mam. | 114152164 | GenBank |
| RnoSTIM-2 | *Rattus norvegicus* | norway rat | Mam. | 109499717 | GenBank |
| CfaSTIM-1 | *Canis familiaris* | dog | Mam. | 73988069 | GenBank |
| CfaSTIM-2 | *Canis familiaris* | dog | Mam. | ENSCAFP00000024080 | Ensembl |
| BtaSTIM-1 | *Bos taurus* | cattle | Mam. | 110757132 | GenBank |
| BtaSTIM-2 | *Bos taurus* | cattle | Mam. | 78369570 | GenBank |
| MmuSTIM-1 | *Macaca mulatta* | Rhesus macaque | Mam. | 109107697 | GenBank |
| MmuSTIM-2 | *Macaca mulatta* | Rhesus macaque | Mam. | 109073940 | GenBank |
| PtrSTIM-1 | *Pan troglodytes* | chimpanzee | Mam. | ENSPTRP00000005665 | Ensembl |
| PtrSTIM-2 | *Pan troglodytes* | chimpanzee | Mam. | ENSPTRP00000027495 | Ensembl |
| HsaSTIM-1 | *Homo sapiens* | human | Mam. | 21070997 | GenBank |
| HsaSTIM-2 | *Homo sapiens* | human | Mam. | 41349446 | GenBank |

Note - Abbreviations: Invt., Invertebrate; Mam., Mammal; Vt., Non-Mammalian Vertebrate. Naming of sequences: We describe all molecules in this study using an abbreviation of genus and species name, followed by names after their phylogenetic relationship with characterized STIM proteins.

***** Sequences that failed in the *Chi-square* test in TREE-PUZZLE or contained more than 15% gaps in the refined alignments were extruded for phylogenetic tree construction.
