# Supplementary figures and images for: Molecular Evolution and Functional Divergence of the Ca2+ Sensor Protein in Store-operated Ca2+ Entry: Stromal Interaction Molecule
Source: PLoS One. 2007 Jul 11;2(7):e609. doi: 10.1371/journal.pone.0000609 (PMC1904252; doi:10.1371/journal.pone.0000609)

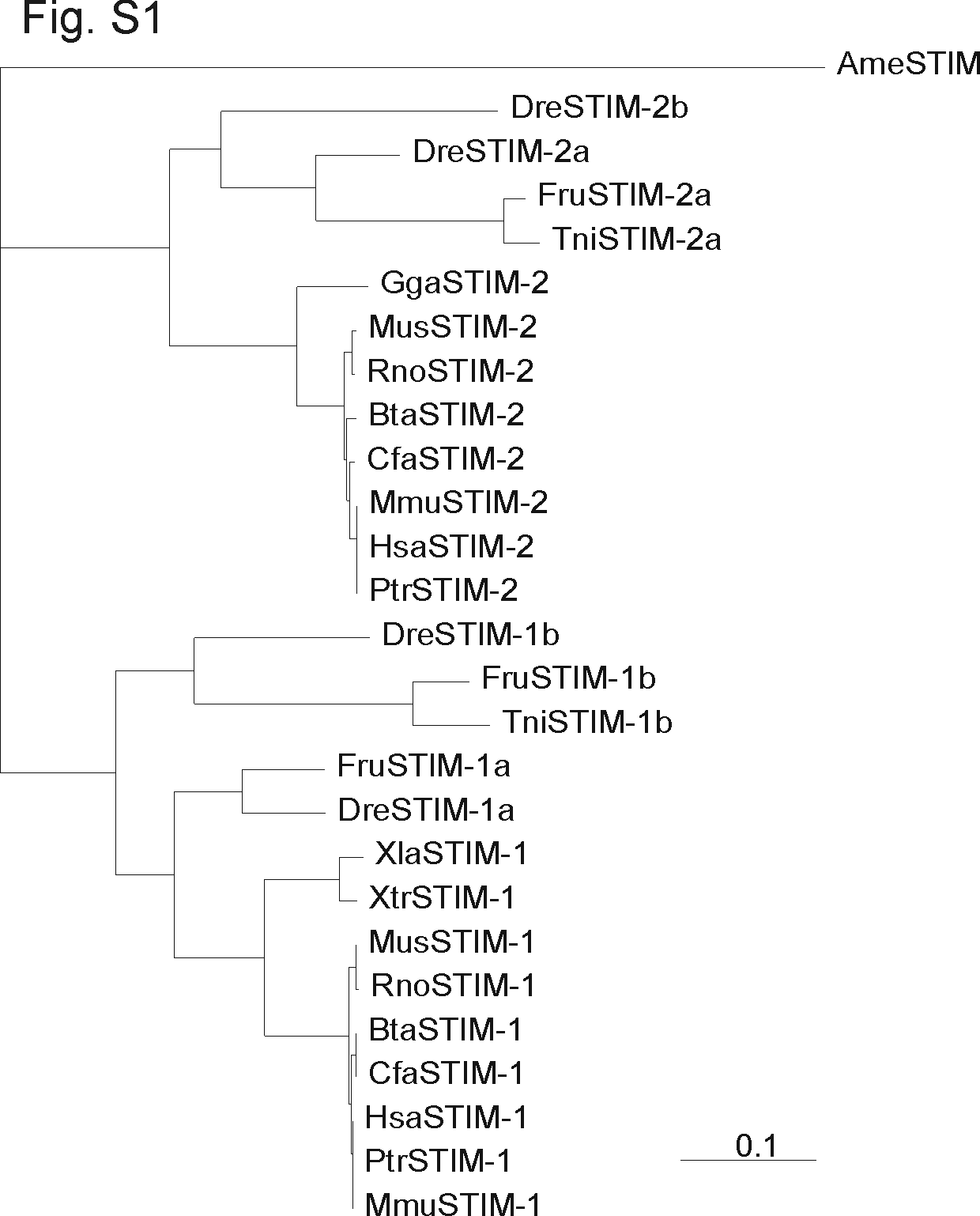

Supplement: Figure S1 — Neighbor-joining tree used for the program DIVERGE. The tree was constructed with Poisson distance matrix implemented in DIVERGE, and re-rooted with the invertebrate STIM sequence AmeSTIM. The two branches for STIM-1 and STIM-2 were then selected as corresponding clusters for subsequent functional divergence analysis between two branches. (1.91 MB TIF) [file pone.0000609.s002.tif]
